# Systematic review on wearable lower-limb exoskeletons for gait training in neuromuscular impairments

**DOI:** 10.1186/s12984-021-00815-5

**Published:** 2021-02-01

**Authors:** Antonio Rodríguez-Fernández, Joan Lobo-Prat, Josep M. Font-Llagunes

**Affiliations:** 1 Biomechanical Engineering Lab, Department of Mechanical Engineering and Research Center for Biomedical Engineering, Universitat Politècnica de Catalunya, Diagonal 647, 08028 Barcelona, Spain; 2 Institut de Recerca Sant Joan de Déu, Santa Rosa 39-57, 08950 Esplugues de Llobregat, Spain; 3 ABLE Human Motion, Diagonal 647, 08028 Barcelona, Spain; 4 Institut de Robòtica i Informàtica Industrial, CSIC-UPC, Llorens i Artigas 4-6, 08028 Barcelona, Spain

**Keywords:** Wearable exoskeleton, Lower-limb, Neuromuscular impairment, Gait rehabilitation, Spinal cord injury, Stroke

## Abstract

Gait disorders can reduce the quality of life for people with neuromuscular impairments. Therefore, walking recovery is one of the main priorities for counteracting sedentary lifestyle, reducing secondary health conditions and restoring legged mobility. At present, wearable powered lower-limb exoskeletons are emerging as a revolutionary technology for robotic gait rehabilitation. This systematic review provides a comprehensive overview on wearable lower-limb exoskeletons for people with neuromuscular impairments, addressing the following three questions: (1) what is the current technological status of wearable lower-limb exoskeletons for gait rehabilitation?, (2) what is the methodology used in the clinical validations of wearable lower-limb exoskeletons?, and (3) what are the benefits and current evidence on clinical efficacy of wearable lower-limb exoskeletons? We analyzed 87 clinical studies focusing on both device technology (e.g., actuators, sensors, structure) and clinical aspects (e.g., training protocol, outcome measures, patient impairments), and make available the database with all the compiled information. The results of the literature survey reveal that wearable exoskeletons have potential for a number of applications including early rehabilitation, promoting physical exercise, and carrying out daily living activities both at home and the community. Likewise, wearable exoskeletons may improve mobility and independence in non-ambulatory people, and may reduce secondary health conditions related to sedentariness, with all the advantages that this entails. However, the use of this technology is still limited by heavy and bulky devices, which require supervision and the use of walking aids. In addition, evidence supporting their benefits is still limited to short-intervention trials with few participants and diversity among their clinical protocols. Wearable lower-limb exoskeletons for gait rehabilitation are still in their early stages of development and randomized control trials are needed to demonstrate their clinical efficacy.

## Background

Gait disorders affect approximately 60% of patients with neuromuscular disorders [1] and generally have a high impact on their quality of life [2]. Moreover, immobility and loss of independence for performing basic activities of daily living results in patients being restricted to a sedentary lifestyle. This lack of physical activity increases the risk of developing secondary health conditions (SHCs), such as respiratory and cardiovascular complications, bowel/bladder dysfunction, obesity, osteoporosis and pressure ulcers [3–7]; which can further reduce the patients’ life expectancy [3, 4]. Therefore, walking recovery is one of the main rehabilitation goals for patients with neuromuscular impairments [8, 9].

Robotic gait rehabilitation appeared 25 years ago as an alternative to conventional manual gait training. Compared with conventional therapy, robotic gait rehabilitation can deliver highly controlled, repetitive and intensive training in an engaging environment [10], reduce the physical burden for the therapist, and provide objective and quantitative assessments of the patients’ progression [11]. The use of gait rehabilitation robots began in 1994 [12] with the development of Lokomat [13]. Since then, different rehabilitation robots have been developed and can be classified into grounded exoskeletons (e.g., Lokomat [14], LOPES [15], ALEX [16]), end-effector devices (e.g., Gait Trainer [17], Haptic Walker [18]), and wearable exoskeletons (e.g., ReWalk [19], Ekso [20], Indego [21]) [12]. In addition, there have been recent developments towards “soft exoskeletons” or “exosuits” which use soft actuation systems and/or structures to assist the walking function [22–25]. Despite these developments, to date the optimal type of rehabilitation robot for a specific user and neuromuscular impairment still remains unclear [26].

Wearable exoskeletons are emerging as revolutionary devices for gait rehabilitation due to both the active participation required from the user, which promotes physical activity [27], and the possibility of being used as an assistive device in the community. The number of studies on wearable exoskeletons during the past 10 years has seen a rapid increase, following the general tendency now towards rehabilitation robots [28]. Some of these devices already have FDA approval and/or CE mark, and are commercially available, whereas many others are still under development.

There have been several reviews surveying the field of wearable exoskeletons for gait rehabilitation. Some of these reviews have focused on reviewing the technological aspects of exoskeletons from a general perspective [29, 30], while others have focused on specific aspects such as the control strategies [31] or the design of specific joints [32]. A selection of reviews have focused on surveying the evidence on effectiveness and usability of exoskeletons for clinical neurorehabilitation in general [33, 34], or for a specific pathology such as spinal cord injury (SCI) [30, 34] or stroke [11].

This review provides a comprehensive overview on wearable lower-limb powered exoskeletons for over ground training, without body weight support, that are intended for use with people who have gait disorders due to neuromuscular impairments. In comparison with other reviews, we analyse a wide range of aspects of wearable exoskeletons, from their technology to their clinical evidence, for different types of pathologies. This systematic review was carried out to address the following questions: (1) what is the current technological status of wearable lower-limb exoskeletons for gait rehabilitation?, (2) what are the benefits and risks for exoskeleton users?, and (3) what is the current evidence on clinical efficacy for wearable exoskeletons?

**Fig. 1:**
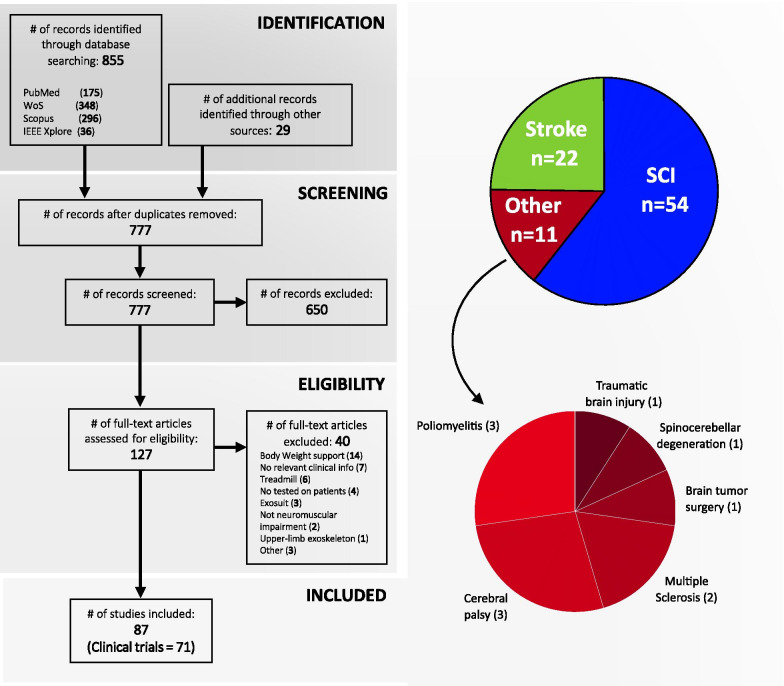
Four-phase flow diagram of the literature selection process according to PRISMA guidelines. From 884, finally 87 studies were selected, of which 71 were identified as clinical trials according to the Clinical Trial definition proposed by the National Institutes of Health (NIH) [35] (see Additional file 1). The 87 studies were grouped in three categories according to the pathology treated in the study: Spinal Cord injury ($\hbox {n}=54$), stroke ($\hbox {n}=22$) and other pathologies ($\hbox {n}=11$; poliomyelitis: 3, cerebral palsy: 3, multiple sclerosis: 2, brain tumor surgery: 1, spinocerebellar degeneration: 1, and traumatic brain injury: 1)

## Methods

### Search strategy

We searched for scientific publications in four online databases from 2000 until 18th March 2019 using the following search terms: (exoskeleton OR orthos* OR exoskeletal) AND (robot* OR power* OR active) AND (walk* OR gait) AND ((leg OR lower) AND (limb OR extremity)) AND (rehabilitation* OR clinical* OR pilot) NOT (“body weight support” OR BWS OR treadmill OR upper OR hand OR arm). This literature search resulted in 855 publications, 57 of which were added in a second search for commercially available exoskeletons: 175 in PubMed, 348 in Web of Science, 296 in Scopus, 36 in IEEE Xplore. Additionally,  29 studies from exoskeleton websites were added.

After removing duplicates, 777 publications were screened first by their title and secondly by their abstract. 127 publications were full-text assessed for eligibility. The identification, screening and eligibility check of the studies were all done by the same author (i.e., A. Rodríguez-Fernández). In case of uncertainty during the screening or the classification process, a decision was reached in agreement with the three authors of the manuscript. Finally, 87 studies were included in this review (Fig. 1), of which 71 were identified as clinical trials according to the Clinical Trial definition proposed by the National Institutes of Health (NIH) [35] (see Additional file 1 for a detailed view on the clinical trial identification assessment). Selected studies were published between 2009 and 2019, focusing this literature study on the last 11 years.

### Inclusion and exclusion criteria

We only included studies written in English, which provided relevant clinical information aimed at studying the effects of exoskeleton devices on gait rehabilitation. To be included in the analysis, each article had to meet the following three conditions: (1) studies had to use a wearable and powered lower-limb exoskeleton, (2) report overground outcome measures, and (3) participants had to have a neuromuscular impairment. There were no limitations regarding the participants’ age or gender. Note that we considered as wearable exoskeletons those that present a rigid external structure and therefore, soft exoskeletons or exosuits were not included in the present survey. Studies that used body weight support or a treadmill were excluded with the purpose of focusing only on studies that solely investigated the effect of wearable exoskeleton technology. Note that for the analysis, only data from patients who used the robotic devices were included, i.e., patients in the intervention group.

### Approach

The information of each study was classified according to technical aspects of the exoskeleton and clinical aspects. The technical aspects included: (1) exoskeleton design and structure, (2) control methods, and (3) type of actuators. The clinical aspects included: (4) patient demographics, (5) patient impairments, (6) training protocol, (7) outcome measures, (8) the walking aids used during training, and (9) the training environment.

The neuromuscular impairments of the patients were classified into three groups: spinal cord injury (SCI), stroke, and other pathologies. This classification was used to analyse the technical and clinical aspects of the 87 studies. Due to the large number of studies involving SCI patients, we carried out a specific analysis on the level of injury (LOI) building upon the previous analysis carried out by Contreras-Vidal et al. [30].

The classification of primary and secondary outcome measures were grouped using the five categories proposed by Contreras-Vidal et al. [30] and a sixth additional category: (1) *Ambulation assessments*, which includes measures to assess locomotor ability based on time or distance measures; (2) *balance and level of assistance/independence*, which evaluates the stability and the dependency on walking aids; (3) *physiological improvements*, which considers effects related to pain, skin, bowel/bladder function and spasticity; (4) *energy expenditure*, which quantifies the effort and metabolic energy consumption needed when using the device; (5) *usability and comfort*, which evaluates the ergonomics and the subjective feedback of the user; and (6) *biomechanics*, which contains the kinematic and kinetic metrics.

Selected studies were grouped in four categories according to their study design: experimental validation (preliminary evaluation of the device), pilot study, observational study (descriptive study, cohort study, longitudinal study, cross-sectional study, pre-post study) and experimental study (randomized control trial).

## Review

### Wearable exoskeleton technology

**Fig. 2:**
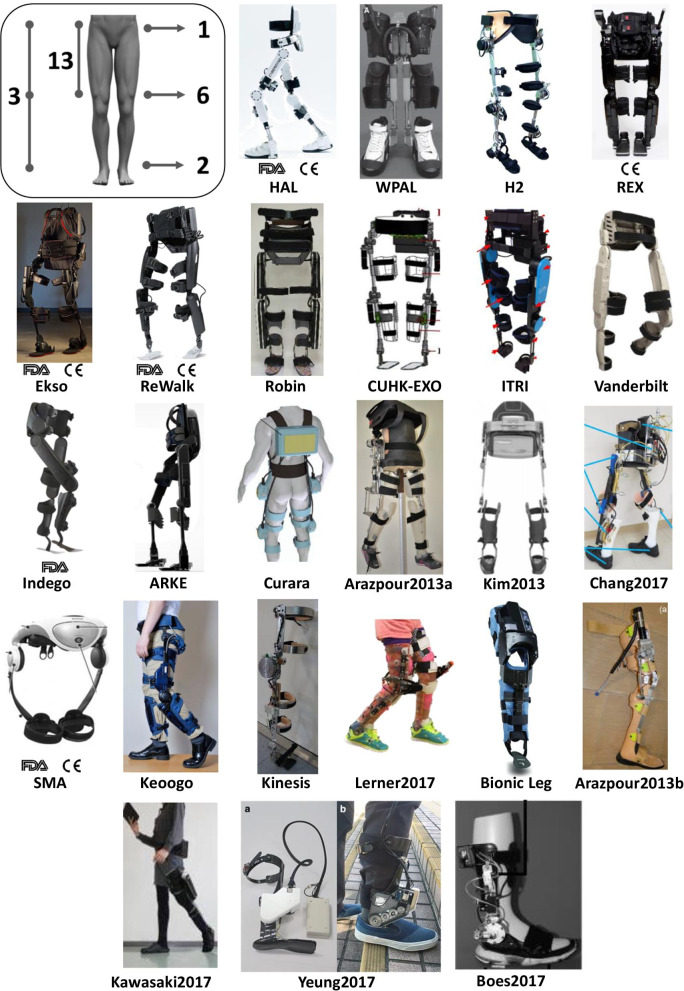
Exoskeletons included in the literature review. From left to right and top to bottom: A diagram showing the locations of the active joints of the exoskeletons included in the literature review, HAL (Image courtesy of Cyberdine, Inc.), WPAL (Reproduced from [39]), H2 (Reproduced from [40]), REX (Reproduced from [41]), Ekso (Image courtesy of Paolo Milia, Prosperius Institute, Neurorehabilitation and Robotic Area, University of Perugia, Umbertide, Italy), ReWalk (Image courtesy of ReWalk Robotics), Robin (Image courtesy of Hyunsub Park, Applied Robot Technology R&D Group, Korea Institute of Industrial Technology, Korea), CUHK-EXO (Reproduced from [42]), ITRI (Reproduced from [43]), Vanderbilt Exoskeleton (Image courtesy of Michael Goldfarb, Vanderbilt University, Nashville), Indego (Reproduced from [44]), ARKE (Image courtesy of Edward Lemaire, Ottawa Hospital Research Institute, Centre for Rehabilitation Research and Development, Ottawa, Canada), Curara (Reproduced from [45]), Arazpour2103a (Image courtesy of Mokhtar Arazpour, Department of orthotics and prosthetics, University of Social Welfare and Rehabilitation Sciences, Tehran, Islamic Republic of Iran), Kim2013 (Image courtesy of Kim Gyoosuk, Korea Workers Compensat & Welf Serv, Rehabil Engn Res Inst, Incheon, South Korea), Chang2017 (Reproduced from [46]), SMA (Reproduced from [47]), Keoogo (Reproduced from [48]), Kinesis (Image courtesy of Antonio J. del Ama, Electronic Technology Deparment, Rey Juan Carlos University, Spain), Lerner2017 (Image courtesy of Thomas Bulea, Rehabilitation Medicine Department, National Institutes of Health Clinical Center, Bethesda, USA), Alter G Bionic Leg (Image courtesy of Luna Solution, S.L.), Arazpour2013b (Image courtesy of Monireh A. Bani, Department of Orthotics and Prosthetics, University of Social Welfare and Rehabilitation Sciences, Tehran, Islamic Republic of Iran), Kawasaki2017 (Image courtesy of Ohata Koji, Department of Human Health Sciences, Kyoto University Graduate School of Medicine, Japan), Yeung2017 (Reproduced from [49]), and Boes2017 (Reproduced from [50]). Note that Vanderbilt Exoskeleton and Kinesis are the former prototypes from the current commercial version of Indego and H2, respectively

This review identified 25 exoskeletons (Fig. 2), from which only six have FDA approval and/or CE mark and are commercially available (i.e. Ekso, HAL, Indego, REX, ReWalk and SMA). We found that 16 out of the 25 exoskeletons (64%) actively assist two or more joints (13: hip-knee, 3: hip-knee-ankle), while the rest (36%) actively assist a single joint (1: hip, 6: knee, 2: ankle). In addition, out of the 25 exoskeletons only one is intended for the paediatric population [36]. Table 1 summarizes the main technical aspects of the 25 exoskeletons. For further details on the exoskeleton characteristics see Additional file 2.

**Fig. 3:**
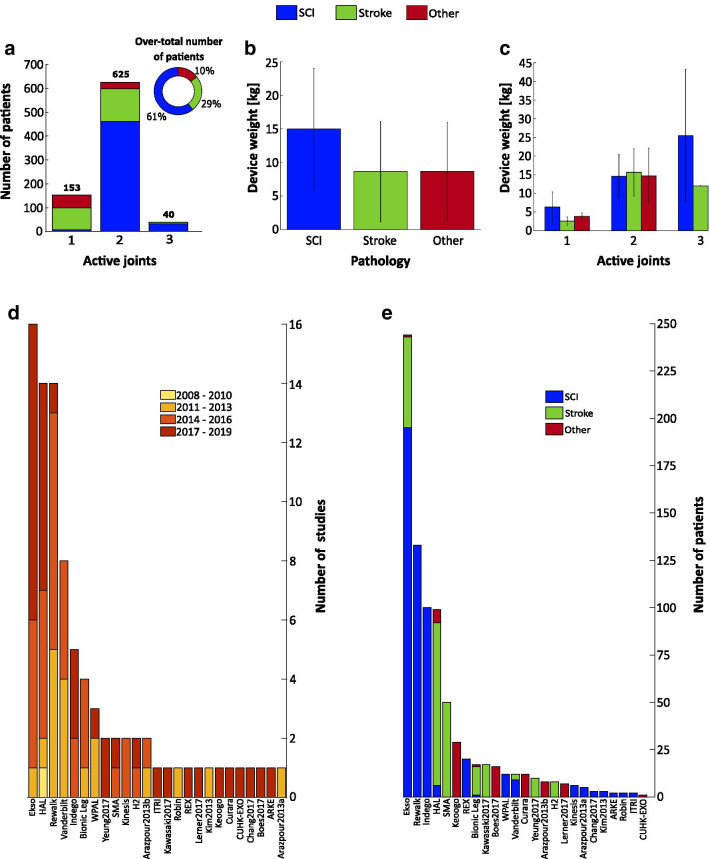
Overview of wearable exoskeletons regarding studied pathologies and number of studies, patients and active joints. **a** Barplot showing the number of patients that have used exoskeletons with 1, 2 or 3 active joints. **b** Barplot showing the weight of wearable exoskeletons for each pathology: spinal cord injury, stroke or other pathologies. **c** Barplot showing the weight of wearable exoskeletons that use 1, 2 or 3 active joints. **d** Number of studies included in this review for each exoskeleton grouped by triennium. **e** Number of patients studied by each exoskeleton grouped by pathology. Error bars indicate one standard deviation

From our literature review, we identified that the first clinical study using a wearable exoskeleton was published in 2009 reporting the results of a clinical test with the HAL exoskeleton [37]. The second study did not appear until 2011 with the clinical evaluation of the Vanderbilt Exoskeleton (nowadays commercialized as Indego) [38]. Moreover, we found that Ekso, HAL and ReWalk are the exoskeletons with a considerably higher number of clinical studies (Fig. 3d), and together with the Indego exoskeleton they have been the most tested exoskeletons in terms of number of patients (Fig. 3e).

#### Design and structure

We found that the number of degrees of freedom (DOF) in wearable exoskeletons ranges from one to three per leg in the sagittal plane (except for REX which also enables movement in the transverse and frontal planes) and the most frequent number of DOF is two (Fig. 2). Joints can be passive, active or, as in the case of the ankle joint, they may also be fixed. From the 25 exoskeletons selected in this review, 22 present an active knee joint (see Table 1), nine present passive joints (8: ankle, 1: hip), 7 present a fixed ankle joint (Indego, ARKE, Arazpour2013a, Arazpour2013b, Kim2013, Chang2017 and AlterG Bionic Leg) and 5 do not present any ankle joint (Vanderbilt Exoskeleton, Curara, SMA, Keoogo and Kawasaki2017).

Exoskeletons with two active joints were tested by 76.4% of the total number of patients reported in the included studies, and focused mostly on SCI patients (Fig. 3a). In contrast, exoskeletons with three active joints were tested by only 4.9% of the patients and also focused on SCI. Finally, exoskeletons with one active joint were tested by 18.7% of the patients and mostly focused on stroke and patients with other pathologies.

In agreement with the trend previously detected by Young and Ferris [51] and Veale and Xie [52], we found that the most frequent actuators are electric motors (22 out of the 25 exoskeletons). Only three of the reviewed exoskeletons use hydraulic [46] or pneumatic actuators [50, 53] (see Table 1). Regarding the power supply, we found that batteries are able to reach up to 6 hours of use in the case of the H2 exoskeleton, but generally they are only capable of sustaining 2 to 4 hours of continuous use (Table 1).

Wearable exoskeletons are still heavy and bulky devices due to their rigid structures, actuators and batteries. For example, the average weight of hip-knee exoskeletons is 14.28 kg (7.14 kg/leg), which approximately corresponds to more than half the weight of an average adult human leg (i.e., 10.88 kg [54]). Note that added loads in the legs result in an increase of the net metabolic cost, and the effect is larger when the load is located more distally [55].

Exoskeletons for SCI patients have the highest mean weight ($15.15 \, \pm \, 9.01\ \hbox {kg}$), independently of the number of active joints (Fig. 3a), mainly due to the fact that the two heaviest exoskeletons were used only in SCI (ReWalk: 23.3 kg, and REX: 38 kg). The mean weight of exoskeletons used in stroke ($8.90 \, \pm \, 7.48\ \hbox {kg}$) and in patients with other pathologies (8.87   ±   7.35 kg) are in the same range. Independently of the pathology, exoskeletons with the same number of active joints have similar weights (Fig. 3c). As expected, we found that there is a relationship between number of active joints and the exoskeleton’s weight: an increase of active joints results in a weight increase.

Studies found that misalignment due to suboptimal fitting can increase the metabolic cost and discomfort of the wearer producing pain, injuries [56, 57] and augment the risk of bone fractures [58, 59]. Therefore, the structure of the exoskeleton has to be able to adapt to the anthropometry of the users [60]. Exoskeletons can adapt to the user’s height with a range of approximately 1.45 to 1.95 m (see Table 1), which covers the majority of the population [61]. However, the maximum allowed weight of 100 kg could be a limiting factor due to the fact that people with neuromuscular impairments present a higher rate of obesity [62, 63]. On the other hand, wearable exoskeletons need to be easy to don/doff in order to prevent users from carrying out hazardous transitions and requiring assistance from caregivers. Doffing time takes around 10 minutes [40, 64, 65] and usually tends to be shorter than donning time, which can reach up to 30 minutes in some cases [66]. In general, patients are unable to don/doff the exoskeleton by themselves [65], often needing to carry out complicated wheelchair-exoskeleton transitions, thus requiring the assistance of caregivers.

Supervision from clinical staff is nearly always required during wearable exoskeletons use. In addition, in order to avoid falls and provide balance, individuals need supportive devices such as crutches, walkers and canes (Fig. 5b), which can limit the independence and mobility of the user, and may lead to shoulder pain [67]. In the study by Manns et al. [68], which evaluated the perspective of the participants after training with the ReWalk exoskeleton, several participants emphasized the effort exerted with the arms while using the exoskeleton. From this review, we found that patients with SCI commonly ended up using a walker or crutches whereas post-stroke patients, due to their hemiparesis, used a cane on the unaffected side. In the group of other pathologies, the walker was the most commonly used aid, and in 4 of these studies no aid was needed.

Soft exoskeletons (or exosuits) have recently arisen to mitigate some of the limitations of conventional, rigid wearable exoskeletons mentioned above. Soft exoskeletons stand out for doing away with rigid frames presented in wearable exoskeletons. Standard soft exoskeletons are characterized for being textile devices actuating on user’s joints through Bowden cable-based transmissions [69, 70]. The soft structure translates into lighter devices which do not restrict the wearer’s mobility, leading to improved comfort, reduced metabolic cost and improved ease to don and doff [69, 71]. However, the low actuation torques prevent soft exoskeletons from assisting people with severe motor impairments, such as non-ambulatory individuals [22, 72].

#### Control and sensing

Wearable exoskeletons started implementing rigid control methods based on predefined trajectories [30]. Nevertheless, exoskeleton technology is opening to patients that are not completely paralyzed and thus, in order to encourage active participation of the user [73] and provide more voluntary control, compliant control methods based on user-exoskeleton interaction (e.g., impedance control) are becoming more frequent (see Table 1). In fact, the study by Pérez-Nombela et al. [74] found that patients with incomplete SCI using the H2 exoskeleton presented higher metabolic cost when they walked with a predefined trajectory than with a control method based on user-exoskeleton interaction. We found that approximately 50% of the included exoskeletons use predefined gait trajectories, and the other 50% implement control methods based on user-exoskeleton interaction. We also found that the HAL exoskeleton is the only device that implements an EMG-based control method [75].

Regardless of the type of control, there are two elements that are crucial for the operation of the exoskeleton: the algorithms for gait phase detection and step initiation (see Table 1). We found that all the exoskeletons included in this review use deterministic threshold-based methods (i.e., a given input will always produce the same output). Despite the limited information provided in studies about this field, we found that the use of ground reaction forces is the most frequent method to detect gait phases (see Table 1), followed by joint angles and inertial measurements. In the cases where the intended users preserve locomotor function, exoskeletons also measure joint torques or EMG signals (see Table 1) generated by the user to trigger steps. Finally, we also found that several exoskeletons use explicit inputs such as buttons or joysticks (see Table 1) to control the exoskeleton.

#### SCI level of injury distribution

**Fig. 4:**
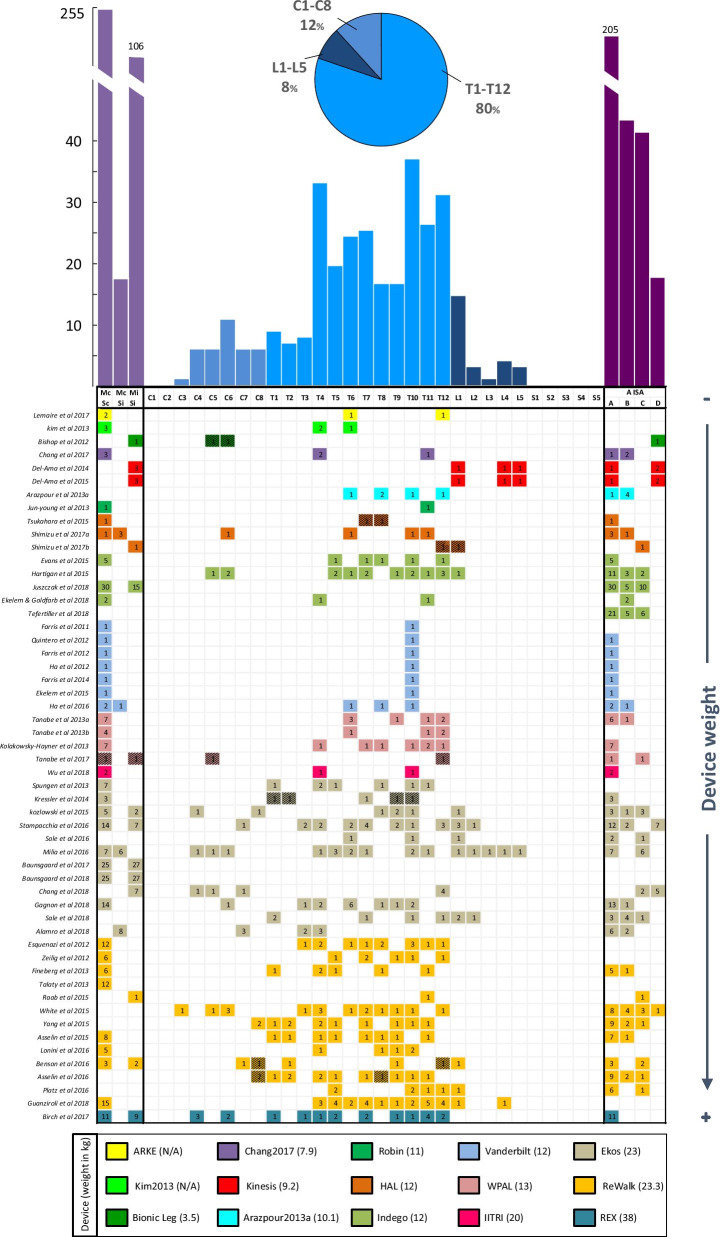
Level of injury (LOI) distribution grouped by exoskeleton and study. The number inside each cell indicates the number of patients that were tested in each study. Colors indicate studies that used the same exoskeleton and are ordered according to the device weight from lightest (top) to heaviest (bottom). Left histogram shows the distribution of patients with lesions that are motor and sensory complete (Mc/Sc), motor and sensory incomplete (Mi/Si) and motor-complete and sensory incomplete (Mc/Si). Middle histogram shows the distribution of patients according to LOI, and the right histogram shows the distribution of patients according to the AISA Impairment Scale (AIS) [88]. Cells with a grated pattern indicate patients that present two different LOI (i.e., patients who have two or more injured vertebrae)

Figure 4 builds upon Figure 1 of Contreras-Vidal et al. [30] and shows the LOI distribution across the clinical studies with SCI patients. In general, the range of LOIs is widely covered from high cervical levels (C3) to low lumbar lesions (L5), yet we did not find studies including patients with LOI of C1, C2, S1, S2, S3, S4 and S5. Patients with thoracic lesions are the most representative (80%) with T10 being the most studied LOI, followed by T4 and T12. The low representation of cervical (12%) and sacral (8%) lesions is probably due to the study inclusion/exclusion criteria, which require patients to be able to use walking aids (e.g., crutches or walkers) and exclude patients that have a low level of walking impairment, i.e., patients with sacral lesions. We found that the Ekso and the ReWalk exoskeletons present the widest range of injuries with the largest number of patients. We also found that exoskeletons without active hip joint are restricted to patients with incomplete or low thoracic-complete LOI.

Figure 4 also shows that approximately 67% of SCI patients have a motor and sensory complete injury (Mc/Sc), 28% have a motor and sensory incomplete injury (Mi/Si), and finally only 18 patients (5%) have a motor-complete sensory-incomplete injury (Mc/Si). This evidence contrasts with data from the National Spinal Cord Injury Statistical Center (NSCISC) where incomplete paraplegia/tetraplegia affects 67.5% of the patients with SCI [76]. The bias detected in the review for complete SCI patients seems to be attributed to the inclusion criteria of the studies. We identified a great number of studies whose only focus was assessing the impact of exoskeletons on motor-complete SCI or non-ambulatory patients, thus excluding anyone who was ambulatory at all. The reason for this inclusion criterion may be due to assist complete SCI subjects with exoskeletons is simpler, especially with control methods based on predefined trajectories. Conversely, if the wearer preserves motor function, the exoskeleton has to cooperate with the subject through user-exoskeleton interaction-based control, which is more complex.

**Table 1:** Main technical aspects of the exoskeletons

Exoskeleton	Actuated joints	Actuator	Sensor	Control method	Gait initiation mode	Device weight (kg)	User height (cm) and weight (kg)	Operation time (h)	Unique features
WPAL [39]	HKA	Electric	JA, JT	Trajectory Interaction	Button	13	145–180 80	$>1$	Alternating use of robot and wheelchair
H2 [40]	HKA	Elecric	JA, JT, IT, FF	Trajectory Interaction	Button	12	145–195 100	6	–
REX [41]	HKA	Electric	–	Trajectory	Joystick	38	146–195 100	1	Joystick and three-button keypad
HAL [37]	HKa$^{*}$	Electric	EMG, JA, FF, Acc	Trajectory Interaction EMG-control	EMG Weight shifts	14	150–190 100	1.5	Independent leg
Ekso [77]	HKa	Electric	JA, FF, Acc AJA, ACF	Trajectory Interaction	Weight shifts Button	23	158–188 100	1	FDA for stroke
ReWalk [19]	HKa	Electric	JA, FF, Ori	Trajectory	Weight shifts CoM (body tilt)	23.3	160–190 100	2	FDA for home use
Robin [78]	HKa	Electric	FF, Acc, CAcc	–	Weight shifts	11	– –	–	–
CUHK-EXO [42]	HKa	Electric	JA, FF, Acc Ori, CF, CAcc	Trajectory	Phone App Crutch buttons Upper body movements	18	155–185 –	3	–
ITRI [43]	HKa	Electric	–	Trajectory	Button	20	– –	–	–
Vanderbilt Exoskeleton [79]	HK	Electric	JA, Acc, Ori	Trajectory Interaction	CoP (body tilt)	12	– –	–	–
Indego [21]	HK$^{\dagger }$	Electric	JA, Acc, Ori	Trajectory Interaction	CoP (body tilt)	12	155–191 113	1.5	FDA for stroke
ARKE [80]	HK$^{\dagger }$	Electric	JA, FF, Acc, Ori	Trajectory	Weight shifts	–	– –	–	–
Curara [45]	HK	Electric	JA, JT, IT	Trajectory Interaction	Motion intent	5.8	– –	–	–
Arazpour2013a [81]	HK$^{\dagger }$	Electric	JA	Trajectory	Orthotist via joystick	10.1	– –	–	–
Kim2013 [53]	HK$^{\dagger }$	Pneumatic	EMG (arms), FF	–	–	–	– –	3	Air muscles for hip
Chang2017 [46]	HK$^{\dagger }$	Hydraulic	JA, FF, Acc, Ori	Trajectory	Button	7.9	152–193 100	2	Functional Neuro-muscular Stimulation
SMA [47]	H	Electric	JA, JT	Trajectory Interaction	Motion intent	2.7	140–200 –	1	–
Keeogo [48]	hK	Electric	–	Trajectory Interaction	Motion intent	5.4	Above 155 –	2.5	Squatting lunging
Kinesis [82]	Ka	Electric	JA, FF, IT, Ori	Trajectory Interaction	Button	9.2	$<185$ 90	–	Hybrid (FES)
Lerner2017 [83]	Ka	Electric	JA, JT, FF	–	–	3.2	Children	1	–
AlterG Bionic Leg [84]	K$^{\dagger }$	Electric	JA, JT, FF, Acc	Trajectory Interaction	Motion intent	3.5	153–182 136	2–3	Unilateral
Arazpour2013b [85, 86]	K$^{\dagger }$	Electric	FF	Trajectory	Weight shifts	3.6	– –	–	Unilateral
Kawasaki2017 [87]	K	Electric	Acc	Trajectory	Motion intent	3	– –	–	Actuator attached to a KAFO Batteries on a belt
Yeung2017 [49]	A	Electric	FF, Acc, Ori	–	Foot lift off	1	– –	5	Battery carried at the waist Unilateral
Boes2017 [50]	A	Pneumatic	JA, FF	Trajectory	Weight shifts	3.1	– –	–	Unilateral

Sensors: Acc: Acceleration; ACF: Arm crutches force; AJA: Arm crutches force; Cacc: crutches acceleration; CF: crutches force/pressure; EMG: electromyography; FF: foot contacting force/pressure; IT: interaction torque; JA: joint angle; JT: joint torque; Ori: orientation; CoM: center of mass; CoP: center of pressure; FES: functional electrical stimulation; KAFO: knee-ankle-foot orthosis

$*$Lowercase letters indicate passive joints

$\dagger$Indicates fixed ankle joint

### Clinical validation

This section analyses the characteristics of the studies including: the study design, the number of patients and their demographics, the training protocol, and the outcome measures used to assess the patients’ performance. An overview of the characteristics in a table format of each of the 87 studies included in this review is available in the Additional file 3. Note that the results described in this section only consider participants who tested the exoskeletons, and not the participants that were in the control group.

#### Study design

Observational studies ($\hbox {n}=35$; 40.2%) represented the most frequent study design among the selected studies, followed by pilot studies ($\hbox {n}=31$; 35.6%), experimental validations ($\hbox {n}=15$; 17.2%) and experimental studies ($\hbox {n}=6$; 6.9%). It should also be pointed out that only 10 out of the 87 studies presented a follow-up evaluation after the intervention with the device [19, 47, 65, 89–95]. The average time elapsed between the study and the follow-up evaluation was 2 months, ranging from 1 week [65] to 1 year [19].

It is clear that there is a lack of experimental studies, since only 6 out of 87 studies included in this review are randomized control trials (RCT). From these RCTs, 5 of them were studies with post-stroke patients [47, 90, 95–97] and the other study focused on people with multiple sclerosis [48]. It should be noted that none of the studies with SCI patients included in this review was a RCT, despite SCI being the most representative impairment (see Fig. 1). Detailed information on study design of the selected studies is available in Additional file 3.

#### Protocol design

**Fig. 5:**
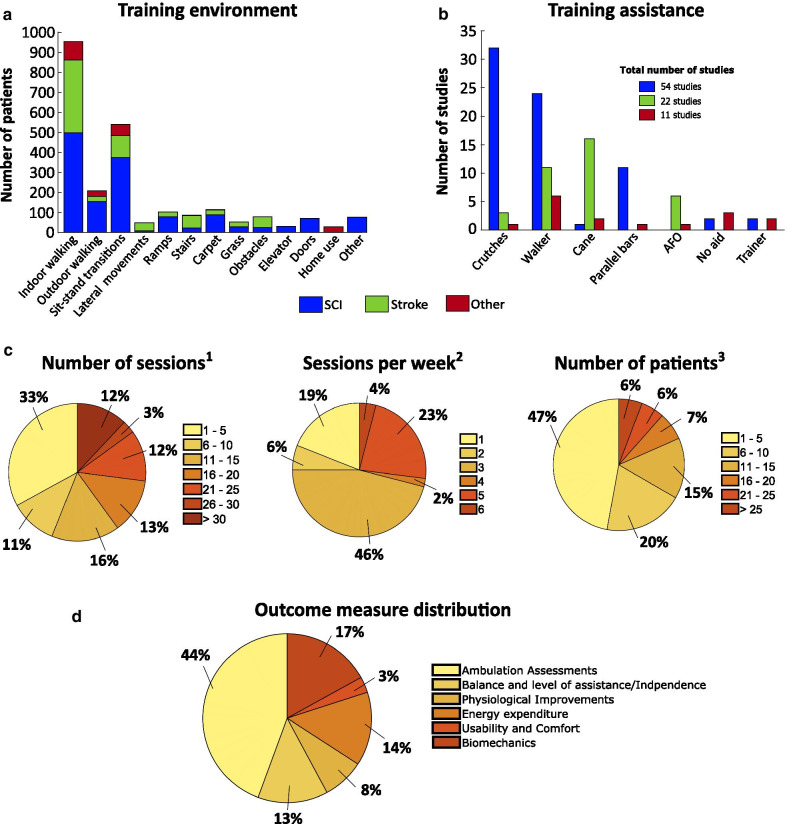
Overview of the study protocol characteristics. **a** Number of patients grouped by pathology for each type of training environment. **b** Number of studies that used supportive devices grouped by pathology. **c** Percentage distribution of number of sessions (left), sessions per week (middle) and number of patients (right) across the selected studies. **d** Percentage distribution of the outcomes measures grouped by categories following the classification done by Contreras-Vidal et al. [30]. $^{1}$82 studies considered, $^{2}$52 studies considered, $^{3}$87 studies considered (only patients from the exoskeleton intervention)

We found that the total number of sessions shows a large variability (range: 1–120), being the range from 1 to 5 sessions the most common (33%). Concerning the number of sessions per week, 3 sessions was the most common frequency (46%) followed by 5 sessions per week (23%; Fig. 5c). Regarding the number of patients, studies with 1 to 5 participants were the most common (47%) with about half of these being single case studies. The maximum number of patients enrolled in one study was 52 [91, 92]. The duration time of each session usually ranged between 60 to 90 minutes, including the donning/doffing time and the rest periods. We found that 4 out of the 87 studies exceed 2 hours per session [36, 41, 44, 98]. Regarding the gender of patients (see Additional file 3), SCI studies show that 79.6% of the patients were males. Despite the large asymmetry, this result agrees with those from the NSCISC, that shows 78% of new cases are male. In post-stroke patients, the percentage of males was also higher (69%) coinciding with stroke worldwide incidence, which is higher among men [99]. Finally, the group of other pathologies presented slightly lower percentage of males (45.3%) than females.

Knowledge about usability of the exoskeleton is a relevant aspect to take into account when developing protocols, since learning to use an exoskeleton is time consuming and variable among users [100]. To date, few studies have focused on the learning process when using exoskeletons [64, 68]. Learning to use an exoskeleton requires not just physical but also mental effort [68]. Kozlowski et al. [64] quantified the time and effort required by people with SCI to learn to use the ReWalk exoskeleton. They found that the average number of sessions (2 hours per session) for walking and developing sit-stand transitions with contact guard assistance (i.e., helper maintains touch or near-touch contact, but provides no assistance) and close supervision were 15 and 18 sessions, respectively. In this regard, there are few studies that showed that the use of biofeedback could accelerate the learning process and reduce the time and effort devoted to learn how to use an exoskeleton [101–104].

As previously concluded by Contreras-Vidal et al. in [30], we found that experimental protocols for clinical validation of exoskeletons present high variability across studies. There is a need for standard clinical guidelines defining protocols for clinical validation of exoskeleton technology. This would also provide the possibility for benchmarking among devices. In this line, the EUROBENCH project aims at establishing standard benchmarking methods for exoskeletons to facilitate comparisons among the available solutions [105, 106].

#### Training protocol

The training protocol shows a common methodology across the selected studies and, in general, studies follow similar methodologies to the one proposed by van Dijsseldonk et al. [107]. In general, after performing the baseline assessment, patients start familiarizing with the device and develop basic skills to use it properly. In this familiarization phase, participants usually practise standing, sitting, balancing and turning. In case patients were not able to do the baseline measurements by themselves (i.e., they were unable to stand up or walk), the “baseline” measurements were taken wearing the exoskeleton in an early stage of the training protocol and the metrics were compared at different time points of the training.

Most of the studies finish the training protocol after a series of indoor walking sessions, yet there are few studies that continue with training more advanced activities such as outdoor walking, stair climbing, walking on different surfaces (carpet, grass, obstacles or ramps), open doors, or elevator use (Fig. 5). We found that, besides indoor walking (done in all the included studies), sit-to-stand transition was the most practised activity, followed by outdoor walking and stair climbing. In some studies, patients received additional training (see Additional file 3) apart from using the exoskeleton. Some of the typical additional training methods used were muscle stretching, balancing activities, range of motion improvement, relaxation and meditation.

#### Outcome measures

Additional file 3 gives an overview of the outcome measures used in the selected studies following the categories proposed by Contreras-Vidal et al. [30], with an additional category that includes metrics related to biomechanics.

We found that outcome measures belonging to the *ambulation assessments* category were the most used (44%), followed by *biomechanics measures* (17%), *energy expenditure* (14%), *balance and level of assistance* (13%), *physiological improvements* (8%), and metrics related to *usability and comfort* (3%; Fig. 5d). We found that the most frequent outcome measures were gait speed (57.5% out of the total number of studies), the 10 meter walk test (10MWT, 43.7%), the 6-minute walk test (6MWT, 43.7%) and the timed up and go test (TUG, 25.3%). Interestingly, all of them belong to the *ambulation assessments* category. The Berg Balance Scale (BBS) was the most common outcome measure of *balance and level of assistance* category, used mainly for stroke patients, although the main outcome measure in stroke studies was the Fugl-Meyer Assessment (FM). Spasticity and pain were the most frequent outcomes in *physiological improvement* category. Moreover, this category, together with *energy expenditure* and *usability and comfort* categories, was mainly focused on people with SCI. In contrast, outcome measures related to *biomechanics* were widely studied independently of the pathology, with knee and hip angles being the most interesting biomechanical outcome measures assessed.

As previously mentioned, outcome measures varied across studies and were mainly focused on aspects related to functional mobility, instead of focusing on analyzing physiological and psychological effects. Only a few studies assessed the improvement related to SHCs. For example, Baunsgaard et al. [92] and Juszczak et al. [108] were the only reviewed studies that have measured bowel/bladder function. They were, together with the study by Jayaraman et al. [97], the only studies that analyzed quality of life, with the latter being the only one accounting for level of depression.

### Benefits and clinical evidence

This section analyses the benefits and risks of using wearable exoskeletons and summarizes the most relevant clinical evidence of this technology. In this section we show the most remarkable information and detailed information can be found in Additional files 3 and 4.

#### Performance assessment

Additional file 3 shows the most common outcome measures used if the study reported an improvement, worsening, no change, or if there was no comparison. In the case of studies focusing on SCI patients, 21 out of 54 studies carried out comparisons of outcome measures. We found that in almost all cases, studies reported an improvement from the first to the last session, which is probably due to the fact that through the training sessions patients adapted to the exoskeleton and learned how to use it. Specifically, in terms of functional mobility, all the studies showed improvements except for two: Bishop et al. [84] showed negative changes in TUG and 10MWT, although the 6MWT did show an improvement; and Chang et al. [109] showed no changes in either gait speed or TUG, although did show improvements in 10MWT and 6MWT. Moreover, we identified three studies that compared the performance of powered exoskeletons with passive knee-ankle-foot orthosis (KAFO) in patients with SCI, and all three showed better results when using the wearable exoskeletons [43, 110, 111].

In contrast to studies with patients with SCI, studies with post-stroke patients assessed the gait performance, without wearing exoskeleton, after training with the exoskeleton and compared the results with the baseline measurements. In general, we found that the degree of mobility improvement was not as substantial as with the studies focusing on SCI patients: 12 out of 16 studies that analyzed gait speed reported an improvement [37, 47, 87, 93, 95–97, 112–116], and only Hassan et al. [117] reported a negative change. The other 3 studies reported no changes. Additionally, 3 out of 9 studies that analyzed Fugl-Meyer scores reported an improvement on the level of the impairment [95, 97, 116]. Regarding the group of studies focusing on other pathologies, 4 out of 7 studies that analyzed outcome measures related to gait speed reported an improvement [36, 86, 118, 119].

#### Clinical evidence

To asses the clinical evidence of wearable exoskeletons as a therapy for walking rehabilitation in people with neuromuscular impairments, we analyzed the results obtained in the aforementioned studies that conducted a RCT (see "Study design" section).

As we have just seen in the previous section, studies focused on people with SCI showed promising results. However, these studies were mainly observational and pilot studies, which implies a questionable evidence. This finding has already been detected in previous works [26, 120]. In the systematic review of Fisahn et al. [120], authors searched for RCTs using exoskeletons as assistive and rehabilitation devices in people with SCI. They identified 11 studies that were RCTs, and 10 of them utilized the robotic exoskeleton Lokomat (grounded exoskeleton). They found no remarkable differences when comparing exoskeleton versus conventional gait therapy. Moreover, the evidence of those studies was low or very low according to the Grading of Recommendations, Assessment, Development and Evaluation (GRADE) System and the risk of bias evaluation conducted by the authors. Similar findings were identified by Mehrholz et al. [26] in their systematic review. Authors found that only 3 out of 9 studies comparing robotic-assisted gait training to conventional overground gait training and other forms of physiotherapy provided usable data. The results obtained in these studies were similar for both training modalities.

Regarding studies with post-stroke patients, we identified 5 RCTs that involved a total of 183 patients. Buesing et al. [47] and Jayaraman et al. [97] compared the SMA exoskeleton versus functional gait training. Significant differences were found in gait variables such as improvements in gait speed, step length and spatial symmetry when using the SMA exoskeleton. Authors also found greater improvements in walking endurance and demonstrated larger changes in corticomotor excitability of the paretic rectus femoris in the SMA group. Watanabe et al. [90], in contrast, did not find significant improvements in either walking speed or stride length when comparing the HAL exoskeleton with conventional gait therapy. However, the HAL group showed a significant improvement in the Functional Ambulation Categories (FAC) test that was maintained at the 2-month follow-up evaluation. Similar results were obtained by Yeung et al. [95] when comparing a powered and a passive version of an AFO. In this case, improvements in FAC test were maintained at the 3-month follow-up evaluation, proving a consistent improvement in gait independence for the group using the powered AFO. Finally, Calabro et al. [96] compared the combination of robotic training with Ekso together with conventional gait training, with conventional gait training alone. The robotic group showed several significant improvements such as gait speed, cortico-spinal excitability and muscle activation, among others. In this line, a Cochrane review [121] concluded that combined treatments (electromechanical-assisted gait training in combination with physiotherapy) after stroke can positively affect gait rehabilitation and are more likely to provide independent walking in post-stroke patients than conventional gait training alone. The same conclusion was reported by Bruni et al. [11] in their systematic review and meta-analysis.

Lastly, we identified one randomized cross over trial in which the authors evaluated the effects of the Keeogo exoskeleton on the physical performance of people with multiple sclerosis, both in a clinical setting and in a home setting [48]. Note that this was the only study from the 87 selected studies that measured the benefits of using a wearable exoskeleton at home. Contrary to what was expected, wearing the Keoogo did not show improvements in physical performance and participants were slower both in walking functional tests (6MWT and TUG) and climbing stairs (Timed Stair Test).

#### Safety and risks

From the 87 studies screened in this review, only 36 provided information on adverse effects derived from the use of wearable exoskeletons. We found only one study [66] reporting falls, which occurred in three patients: two of them when they were starting to ambulate with forearm crutches, and the other patient fell down during a sit-to-stand transition (because of mechanical programming errors as mentioned in the original study). A total of 18 studies reported mild to moderate adverse events such as orthostatic hypertension [122, 123], skin abrasions [21, 48, 64–66, 89, 91, 96, 109, 124–128], fatigue of the upper extremities [123, 127], low back pain [66, 92], and other adverse events such as urinary tract infections [126], talus fracture [126], dizziness [91], calcaneus fracture [123] and severe knee hyperextension [123]. Studies also described that skin abrasions were reduced using padding and size adjustments, and that fatigue of the upper extremities improved with practice.

Despite the fact that, in general, studies show that wearable exoskeletons are safe devices, these results may not be fully representative. According to He et al. [58], studies tend to omit relevant details when reporting adverse events, differ on the inclusion/exclusion criteria, and do not report explicitly whether adverse events occurred. In the study by van Herpen et al. [59], the authors reported the occurrence of two cases of bone fractures during training with exoskeleton and provided instructions for handling accidental situations such as an unexpected shut down of the control system of the exoskeleton.

### Limitations

In this review, we did not use delimiters related to study design nor assessed the study quality. The lack of delimiters could produce some bias, especially for the conclusions related to clinical effectiveness of wearable lower-limb exoskeletons. However, we tried to mitigate this bias by focusing only on experimental studies (i.e., RCT) when discussing the clinical evidence of wearable exoskeletons. Nonetheless, the main aim of this review was to provide a comprehensive overview of wearable lower-limb exoskeletons for clinical applications, so we considered that displaying all the literature without limiting by study design would provide a broader view of the topic.

## Conclusions

In this paper we reviewed the design and clinical evaluation of wearable lower-limb exoskeletons intended to support walking in people with neuromuscular impairments. Since its nascence around 20 years ago, the field of wearable exoskeletons has shown significant progress at supporting the walking function for individuals with neuromuscular impairments. However, it is still challenged by its small evidence base, slow acceptability, complex technical problems and inordinate costs for purchasing. We conclude this review paper by summarizing the main conclusions for each of the proposed research questions.

### What is the current status of wearable lower-limb powered exoskeleton technology for gait rehabilitation?

Wearable exoskeletons are still heavy and bulky devices that in general require supervision (usually from clinical staff) and the use of walking aids, which hinders mobility and independence. All the reviewed exoskeletons use deterministic gait phase detection algorithms following button press or a threshold-based approach. For the latter, foot-ground contact force measurement through insole sensors is the most common metric used. The most frequent type of measurement in wearable exoskeletons is joint angle, since the vast majority of actuators are used together with encoders or potentiometers to provide position feedback. Regarding actuation, the most frequent actuators are electric motors, probably due to the fact that they are easy to control and exhibit great precision with high specific power [52]. Control methods based on predefined trajectories were the first ones to be implemented in wearable exoskeletons [30]. Nevertheless, control methods based on user-exoskeleton interaction, which require a more active participation of the user, are becoming more frequent for rehabilitation purposes. Regarding ergonomic aspects, complex mechanical structures increase the exoskeleton donning/doffing time, which ranges from 10 (doffing) to 30 (donning) minutes. Additionally, joint misalignment is still an issue in current exoskeletons, which may increase metabolic cost and discomfort of the wearer, and it could even generate skin abrasions, ulcers and an increase risk of fractures.

Wearable exoskeletons need to progress towards modular systems capable of adapting to the user’s motor capabilities and limitations. In the same way, control methods should be based on Assist-As-Needed algorithms to conveniently adapt actuation to the user needs according to the rehabilitation process. Moreover, neuronal technology may have an important role for the next generation of wearable exoskeletons. Brain machine interfaces (BMI) allow direct and voluntary control of the devices irrespective of the user capabilities [129] which could enhance the control of exoskeletons [130]. Wearable exoskeletons are intended to be used as assistive devices in daily living activities such as climbing stairs, walking on different surfaces, entering cars and side stepping [131]; however, these functions are poorly covered by current exoskeletons. Finally, the cost of wearable exoskeletons for personal use must be reduced, since their current costs are still prohibitive for the general population [132]. In fact, in the study by Manns et al. [68] nine out of eleven participants said that they would be willing to take the exoskeleton home if the cost of the device was not a factor.

### What is the methodology used in the clinical validations of wearable lower-limb exoskeletons?

Clinical validation studies of wearable exoskeletons are currently in their early stages, thus evidence is still limited to short intervention trials with few participants, as it was concluded in a previous study by Mekki et al. [133]. Study designs are mainly focused on observational studies and pilot studies, thus more efforts should be made in conducting experimental studies with control groups to obtain stronger evidence on clinical effectiveness.

Protocol design and outcome measures vary across studies, which hinders their comparison. Outcome measures, despite presenting encouraging results, are mainly focused on ambulation assessments (i.e., 10MWT, 6MWT, TUG) rather than being centered on physiological and psychological changes to improve or avoid SHCs. Since prevention of SHCs is a primary aim, especially in SCI [134], studies assessing robotic gait therapy with wearable exoskeletons should focus more on outcome measures related to SHCs.

### What are the benefits and the current evidence of clinical efficacy for wearable lower-limb exoskeletons?

Robotic therapy is progressing toward wearable exoskeletons since they offer the advantages of grounded exoskeletons, as well as providing more active participation of the user. Wearable exoskeletons offer the opportunity to socialize more easily with the environment, increasing quality of life and decreasing depression rate [19, 92, 108, 135]. Likewise, standing has plenty of health benefits such as improved blood circulation, reflex activity, and bowel and bladder function [136]. In addition, there are many psychological and social benefits associated with standing, including improved self-image, eye-to-eye interpersonal contact, and daily living independence [137]. All these benefits favour mainly non-ambulatory patients. In fact, we found that patients with SCI are currently the main users of this technology. Nevertheless, studies carried out in post-stroke patients are the ones that present the most reliable and promising results in terms of rehabilitation efficacy in favour of robotic training over conventional gait therapy [11, 121].

Despite the previous benefits, the optimal type of rehabilitation robot for a specific patient’s needs still remains unclear [138–140]. Literature comparing overground wearable exoskeletons with other types of gait therapy is still scarce, especially in people with SCI. Therefore, randomized control trials, comparing overground wearable exoskeletons with other types of robotic gait therapy or conventional gait therapy, are needed to demonstrate both their effectiveness as a rehabilitation device and their impact in psychological and physiological SHCs.

In any case, overground wearable exoskeletons stand out for providing more movement freedom during gait, the opportunity of independent training at home, and the possibility to carry out more activities of daily living such as sitting, turning and climbing stairs. These advantages activate mechanisms of neural plasticity and connectivity re-modulation [96, 141]; which have been proposed as the main factors promoting motor function recovery in SCI and stroke patients [96, 142]. However, although results show that wearable exoskeletons are generally safe devices [143], there is always the risk of unforeseen serious adverse events [59]. Thus, more efforts are needed to develop adequate standards and regulations to have a better understanding of the adverse events and risks of using wearable exoskeletons [58].

In conclusion, efforts should be invested in developing lightweight and easy-to-use exoskeletons, which should be validated through well-defined protocols to provide the best patient-specific rehabilitation training and offer the possibility of benchmarking.

### Recommendations for future research and development

- Size and weight of wearable exoskeletons should be reduced, and structures should be simplified to allow independent donning/doffing and transportability, while increasing user acceptance.
- Balance capabilities of wearable exoskeletons should be improved to reduce the use of supportive devices.
- Control methods should focus on Assist-As-Needed control algorithms to conveniently adapt assistance to the user needs, increase active participation and promote neural plasticity.
- Studies need standard clinical guidelines that define protocols for clinical validation, and regulations to have a better understanding of the adverse events and risks of using wearable exoskeletons.
- Research studies should focus more on outcome measures related to SHCs, since prevention of secondary health problems is a primary aim in rehabilitation.
- Randomized control trials are needed to demonstrate clinical efficacy of wearable exoskeletons when comparing with conventional gait therapy and/or other types of robotic gait therapy, since most of the literature is based on observational and pilot studies.

## Supplementary information

**Additional file 1.** Clinical trial identification assessment.

**Additional file 2.** Wearable lower-limb exoskeletons.

**Additional file 3.** Clinical evidence of wearable lower-limb exoskeletons.

**Additional file 4.** Outcomes measures.

## Data Availability

All data generated or analysed during this study are included in this published article and its supplementary information files.

## Abbreviations

Acc: Acceleration
ACF: Arm crutches force
AIS: AISA Impairment Scale
AJA: Arm crutches force
BBS: Berg balance score
BMI: Brain machine interface
Cacc: Crutches acceleration
CF: Crutches force/pressure
CoM: Center of mass
CoP: Center of pressure
DOF: Degrees of freedom
EMG: Electromyography
FES: Functional electrical stimulation
FF: Foot contacting force/pressure
FM: Fugl-Meyer assessment
IT: Interaction torque
JA: Joint angle
JT: Joint torque
KAFO: Knee-ankle-foot orthosis
LOI: Level of injury
Mc/Sc: Motor and sensory complete injury
Mc/Si: Motor-complete sensory-incomplete injury
Mi/Si: Motor and sensory incomplete injury
N/A: Not available
NIH: National Institutes of Health
NSCISC: National Spinal Cord Injury Statistical Center
Ori: Orientation
RCT: Randomized Control Trial
SCI: Spinal cord injury
TUG: Timed up and go test
10MWT: 10 meter walk test
6MWT: 6 minute walk test

## References

[1] Stolze H, Klebe S, Baecker C, Zechlin C, Friege L, Pohle S, Deuschl G (2005). Prevalence of gait disorders in hospitalized neurological patients. Movem Disord.

[2] Mahlknecht P, Kiechl S, Bloem BR, Willeit J, Scherfler C, Gasperi A, Rungger G, Poewe W, Seppi K (2013). Prevalence and burden of gait disorders in elderly men and women aged 60–97 years: a population-based study. PLoS ONE.

[3] Booth FW, Roberts CK, Laye MJ (2011). Lack of exercise is a major cause of chronic diseases. Comprehens Physiol.

[4] Knight JA (2012). Physical inactivity: associated diseases and disorders. Ann Clin Lab Sci.

[5] Baylor C, Yorkston KM, Jensen MP, Truitt AR, Molton IR (2014). Scoping review of common secondary conditions after stroke and their associations with age and time post stroke. Topics Stroke Rehabil.

[6] Sezer N, Akkuş S, Uğurlu FG (2015). Chronic complications of spinal cord injury. World J Orthop.

[7] Jensen M, Truitt A, Schomer K, Yorkston K, Baylor C, Molton I (2013). Frequency and age effects of secondary health conditions in individuals with spinal cord injury: a scoping review. Spinal Cord.

[8] Harris JE, Eng JJ (2004). Goal priorities identified through client-centred measurement in individuals with chronic stroke. Physiother Can.

[9] Ditunno P, Patrick M, Stineman M, Ditunno J (2008). Who wants to walk? preferences for recovery after sci: a longitudinal and cross-sectional study. Spinal Cord.

[10] Carpino G, Pezzola A, Urbano M, Guglielmelli E (2018). Assessing effectiveness and costs in robot-mediated lower limbs rehabilitation: a meta-analysis and state of the art. J Healthc Eng.

[11] Bruni MF, Melegari C, De Cola MC, Bramanti A, Bramanti P, Calabrò RS (2018). What does best evidence tell us about robotic gait rehabilitation in stroke patients: a systematic review and meta-analysis. J Clin Neurosci.

[12] Gassert R, Dietz V (2018). Rehabilitation robots for the treatment of sensorimotor deficits: a neurophysiological perspective. J Neuroeng Rehabil.

[13] Colombo G, Joerg M, Schreier R, Dietz V (2000). Treadmill training of paraplegic patients using a robotic orthosis. J Rehabil Res Dev.

[14] Colombo G, Wirz M, Dietz V (2001). Driven gait orthosis for improvement of locomotor training in paraplegic patients. Spinal Cord.

[15] Veneman JF, Kruidhof R, Hekman EE, Ekkelenkamp R, Van Asseldonk EH, Van Der Kooij H (2007). Design and evaluation of the lopes exoskeleton robot for interactive gait rehabilitation. IEEE Trans Neural Syst Rehabil Eng.

[16] Banala SK, Kim SH, Agrawal SK, Scholz JP (2008). Robot assisted gait training with active leg exoskeleton (alex). IEEE Trans Neural Syst Rehabil Eng.

[17] Hesse S, Uhlenbrock D (2000). A mechanized gait trainer for restoration of gait. J Rehabil Res Dev.

[18] Schmidt H, Werner C, Bernhardt R, Hesse S, Krüger J (2007). Gait rehabilitation machines based on programmable footplates. J Neuroeng Rehabili.

[19] Esquenazi A, Talaty M, Packel A, Saulino M (2012). The rewalk powered exoskeleton to restore ambulatory function to individuals with thoracic-level motor-complete spinal cord injury. Am J Phys Med Rehabil.

[20] Strickland E (2012). Good-bye, wheelchair. IEEE Spectrum.

[21] Hartigan C, Kandilakis C, Dalley S, Clausen M, Wilson E, Morrison S, Etheridge S, Farris R (2015). Mobility outcomes following five training sessions with a powered exoskeleton. Topics Spinal Cord Injury Rehabil.

[22] Awad LN, Bae J, O’donnell K, De Rossi SM, Hendron K, Sloot LH, Kudzia P, Allen S, Holt KG, Ellis TD (2017). A soft robotic exosuit improves walking in patients after stroke. Sci Transl Med.

[23] Schmidt K, Duarte JE, Grimmer M, Sancho-Puchades A, Wei H, Easthope CS, Riener R (2017). The myosuit: Bi-articular anti-gravity exosuit that reduces hip extensor activity in sitting transfers. Front Neurorobot.

[24] Bae J, Siviy C, Rouleau M, Menard N, O’Donnell K, Geliana I, Athanassiu M, Ryan D, Bibeau C, Sloot L, et al. A lightweight and efficient portable soft exosuit for paretic ankle assistance in walking after stroke. In: 2018 IEEE international conference on robotics and automation (ICRA); 2018. pp. 2820–2827. IEEE.

[25] Sposito M, Poliero T, Di Natali C, Ortiz J, Pauli C, Graf E, De Eyto A, Bottenberg E, Caldwell D. Evaluation of xosoft beta-1 lower limb exoskeleton on a post stroke patient. In: Sixth national congress of bioengineering, Milan, Italy 25-27 June 2018 2018.

[26] Mehrholz J, Harvey LA, Thomas S, Elsner B (2017). Is body-weight-supported treadmill training or robotic-assisted gait training superior to overground gait training and other forms of physiotherapy in people with spinal cord injury? A systematic review. Spinal Cord.

[27] Buchholz AC, McGillivray CF, Pencharz PB (2003). Physical activity levels are low in free-living adults with chronic paraplegia. Obes Res.

[28] Esquenazi A, Talaty M (2019). Robotics for lower limb rehabilitation. Phys Med Rehabil Clin N Am.

[29] Rupal BS, Rafique S, Singla A, Singla E, Isaksson M, Virk GS (2017). Lower-limb exoskeletons: research trends and regulatory guidelines in medical and non-medical applications. Int J Adv Robot Syst.

[30] Contreras-Vidal JL, A Bhagat N, Brantley J, Cruz-Garza JG, He Y, Manley Q, Nakagome S, Nathan K, Tan SH, Zhu F, Pons JL (2016). Powered exoskeletons for bipedal locomotion after spinal cord injury. J Neural Eng.

[31] Meng W, Liu Q, Zhou Z, Ai Q, Sheng B, Xie SS (2015). Recent development of mechanisms and control strategies for robot-assisted lower limb rehabilitation. Mechatronics.

[32] Shorter KA, Xia J, Hsiao-Wecksler ET, Durfee WK, Kogler GF (2013). Technologies for powered ankle-foot orthotic systems: possibilities and challenges. IEEE/ASME Trans Mech.

[33] Federici S, Meloni F, Bracalenti M, De Filippis ML (2015). The effectiveness of powered, active lower limb exoskeletons in neurorehabilitation: a systematic review. NeuroRehabilitation.

[34] Lajeunesse V, Vincent C, Routhier F, Careau E, Michaud F (2015). Exoskeletons’ design and usefulness evidence according to a systematic review of lower limb exoskeletons used for functional mobility by people with spinal cord injury. Disabil Rehabil Assis Technol.

[35] NIH’s Definition of a Clinical Trial | grants.nih.gov. https://grants.nih.gov/policy/clinical-trials/definition.htm. Accessed 06 Mar 2020.

[36] Lerner ZF, Damiano DL, Bulea TC (2017). A lower-extremity exoskeleton improves knee extension in children with crouch gait from cerebral palsy. Sci Transl Med.

[37] Kawamoto H, Hayashi T, Sakurai T, Eguchi K, Sankai Y. Development of single leg version of hal for hemiplegia. In: 2009 annual international conference of the IEEE engineering in medicine and biology society; IEEE. 2009. pp. 5038–43. 10.1109/IEMBS.2009.5333698. 10.1109/IEMBS.2009.5333698 19964376

[38] Farris RJ, Quintero HA, Goldfarb M, Quintero HA, Goldfarb M, Farris RJ, Goldfarb M (2011). Preliminary evaluation of a powered lower limb orthosis to aid walking in paraplegic individuals. IEEE Trans Neural Syst Rehabil Eng.

[39] Tanabe S, Hirano S, Saitoh E (2013). Wearable Power-Assist Locomotor (WPAL) for supporting upright walking in persons with paraplegia. NeuroRehabilitation.

[40] Bortole M, Venkatakrishnan A, Zhu F, Moreno JC, Francisco GE, Pons JL, Contreras-Vidal JL (2015). The H2 robotic exoskeleton for gait rehabilitation after stroke: early findings from a clinical study. J NeuroEng Rehabil.

[41] Birch N, Graham J, Priestley T, Heywood C, Sakel M, Gall A, Nunn A, Signal N (2017). Results of the first interim analysis of the RAPPER II trial in patients with spinal cord injury: ambulation and functional exercise programs in the REX powered walking aid. J NeuroEng Rehabil.

[42] Chen B, Zhong C-H, Zhao X, Ma H, Guan X, Li X, Liang F-Y, Cheng JCY, Qin L, Law S-W, Liao W-H (2017). A wearable exoskeleton suit for motion assistance to paralysed patients. J Orthopaedic Transl.

[43] Wu C-H, Mao H-F, Hu J-S, Wang T-Y, Tsai Y-J, Hsu W-L (2018). The effects of gait training using powered lower limb exoskeleton robot on individuals with complete spinal cord injury. J NeuroEng Rehabil.

[44] Ekelem A, Goldfarb M (2018). Supplemental stimulation improves swing phase kinematics during exoskeleton assisted gait of sci subjects with severe muscle spasticity. Front Neurosci.

[45] Tsukahara A, Yoshida K, Matsushima A, Ajima K, Kuroda C, Mizukami N, Hashimoto M (2018). Effects of gait support in patients with spinocerebellar degeneration by a wearable robot based on synchronization control. J NeuroEng Rehabil.

[46] Chang SR, Nandor MJ, Li L, Kobetic R, Foglyano KM, Schnellenberger JR, Audu ML, Pinault G, Quinn RD, Triolo RJ (2017). A muscle-driven approach to restore stepping with an exoskeleton for individuals with paraplegia. J NeuroEng Rehabil.

[47] Buesing C, Fisch G, O’Donnell M, Shahidi I, Thomas L, Mummidisetty CK, Williams KJ, Takahashi H, Rymer WZ, Jayaraman A (2015). Effects of a wearable exoskeleton stride management assist system (SMA®) on spatiotemporal gait characteristics in individuals after stroke: a randomized controlled trial. J NeuroEng Rehabil.

[48] McGibbon CA, Sexton A, Jayaraman A, Deems-Dluhy S, Gryfe P, Novak A, Dutta T, Fabara E, Adans-Dester C, Bonato P (2018). Evaluation of the Keeogo exoskeleton for assisting ambulatory activities in people with multiple sclerosis: an open-label, randomized, cross-over trial. J NeuroEng Rehabil.

[49] Yeung L-F, Ockenfeld C, Pang M-K, Wai H-W, Soo O-Y, Li S-W, Tong K-Y. Design of an exoskeleton ankle robot for robot-assisted gait training of stroke patients. In: 2017 international conference on rehabilitation robotics (ICORR); IEEE. 2017. pp. 211–215. 10.1109/ICORR.2017.8009248. 10.1109/ICORR.2017.8009248 28813820

[50] Shorter KA, Kogler GF, Loth E, Durfee WK, Hsiao-Wecksler ET (2011). A portable powered ankle-foot orthosis for rehabilitation. J Rehabil Res Dev.

[51] Young AJ, Ferris DP (2017). State of the art and future directions for lower limb robotic exoskeletons. IEEE Trans Neural Syst Rehabil Eng.

[52] Veale AJ, Xie SQ (2016). Towards compliant and wearable robotic orthoses: a review of current and emerging actuator technologies. Med Eng Phys.

[53] Kim G, Kang S, Kang S, Ryu J, Mun M, Kim K (2009). Unlockable knee joint mechanism for powered gait orthosis. Int J Precis Eng Manufact.

[54] Plagenhoef S, Gaynor Evans F, Abdelnour T (1983). Anatomical data for analyzing human motion. Res Q Exer Sport.

[55] Browning RC, Modica JR, Kram R, Goswami A (2007). The effects of adding mass to the legs on the energetics and biomechanics of walking. Med Sci Sports Exer.

[56] Schiele A. Fundamentals of Ergonomic Exoskeleton Robots. Ph.D. thesis 2008.

[57] Näf MB, Junius K, Rossini M, Rodriguez-Guerrero C, Vanderborght B, Lefeber D (2018). Misalignment compensation for full human-exoskeleton kinematic compatibility: State of the art and evaluation. Appl Mech Rev.

[58] He Y, Eguren D, Luu TP, Contreras-Vidal JL (2017). Risk management and regulations for lower limb medical exoskeletons: a review. Med Devices Evid Res.

[59] van Herpen FHM, van Dijsseldonk RB, Rijken H, Keijsers NLW, Louwerens JWK, van Nes IJW (2019). Case report: description of two fractures during the use of a powered exoskeleton. Spinal Cord Series Cases.

[60] Onen U, Botsali FM, Kalyoncu M, Tinkir M, Yilmaz N, Sahin Y (2014). Design and actuator selection of a lower extremity exoskeleton. IEEE/ASME Trans Mech.

[61] Roser M, Appel C, Ritchie H. Human height. Our world in data. 2013.

[62] Liou T-H, Pi-Sunyer FX, Laferrere B (2005). Physical disability and obesity. Nutr Rev.

[63] Gorgey A, Gater D (2007). Prevalence of obesity after spinal cord injury. Topics Spinal Cord Injury Rehabil.

[64] Kozlowski A, Bryce T, Dijkers M (2015). Time and effort required by persons with spinal cord injury to learn to use a powered exoskeleton for assisted walking. Topics Spinal Cord Injury Rehabil.

[65] Tefertiller C, Hays K, Jones J, Jayaraman A, Hartigan C, Bushnik T, Forrest GF (2018). Initial outcomes
from a multicenter study utilizing the indego powered exoskeleton in spinal
cord injury. Topics Spinal Cord Injury Rehabil.

[66] Kolakowsky-Hayner SA, Crew J, Moran S, Shah A (2013). Safety and feasibility of using the eksotm bionic exoskeleton to aid ambulation after spinal cord injury. J Spine.

[67] Jain NB, Higgins LD, Katz JN, Garshick E (2010). Association of shoulder pain with the use of mobility devices in persons with chronic spinal cord injury. PMR.

[68] Manns PJ, Hurd C, Yang JF (2019). Perspectives of people with spinal cord injury learning to walk using a powered exoskeleton. J NeuroEng Rehabil.

[69] Asbeck AT, De Rossi SM, Holt KG, Walsh CJ (2015). A biologically inspired soft exosuit for walking assistance. Int J Robot Res.

[70] Panizzolo FA, Galiana I, Asbeck AT, Siviy C, Schmidt K, Holt KG, Walsh CJ (2016). A biologically-inspired multi-joint soft exosuit that can reduce the energy cost of loaded walking. J Neuroeng Rehabil.

[71] Sanchez-Villamañan MDC, Gonzalez-Vargas J, Torricelli D, Moreno JC, Pons JL (2019). Compliant lower limb exoskeletons: a comprehensive review on mechanical design principles. J NeuroEng Rehabil.

[72] Yang X, She H, Lu H, Fukuda T, Shen Y (2017). State of the art: bipedal robots for lower limb rehabilitation. Appl Sci.

[73] Durandau G, Farina D, Asín-Prieto G, Dimbwadyo-Terrer I, Lerma-Lara S, Pons JL, Moreno JC, Sartori M (2019). Voluntary control of wearable robotic exoskeletons by patients with paresis via neuromechanical modeling. J Neuroeng Rehabil.

[74] Pérez-Nombela S, Del-Ama AJ, Asín-Prieto G, Piñuela-Martín E, Lozano-Berrio V, Serrano-Muñoz D, Gil-Agudo Á, Pons JL, Moreno JC. Physiological evaluation of different control modes of lower limb robotic exoskeleton h2 in patients with incomplete spinal cord injury. In: Converging clinical and engineering research on neurorehabilitation II; 2017. pp. 343–348. Springer. 10.1007/978-3-319-46669-9_58

[75] CYBERDYNE. HAL (Hybrid Assistive Limb). https://www.cyberdyne.jp/english/products/HAL/index.html. Accessed 9 Mar 2020.

[76] NSCISC 2019. National Spinal Cord Injury Statistical Center Facts and Figures at a Glance. https://www.nscisc.uab.edu/Public/FactsandFigures2019-Final.pdf Accessed 2 May 2020.

[77] Strausser KA, Swift TA, Zoss AB, Kazerooni H, Bennett BC. Mobile exoskeleton for spinal cord injury: Development and testing. In: ASME 2011 dynamic systems and control conference and bath/ASME symposium on fluid power and motion control. American Society of Mechanical Engineers Digital Collection. 2011. pp. 419–425. 10.1115/DSCC2011-6042.

[78] Jung J, Jang I, Riener R, Park H (2012). Walking intent detection algorithm for paraplegic patients using a robotic exoskeleton walking assistant with crutches. International Journal of Control, Automation and Systems.

[79] Quintero HA, Farris RJ, Goldfarb M. Control and implementation of a powered lower limb orthosis to aid walking in paraplegic individuals. In: 2011 IEEE international conference on rehabilitation robotics; IEEE. 2011. pp. 1–6. 10.1109/ICORR.2011.5975481. 10.1109/ICORR.2011.5975481 PMC3402219 22275679

[80] Lemaire ED, Smith AJ, Herbert-Copley A, Sreenivasan V (2017). Lower extremity robotic exoskeleton training: case studies for complete spinal cord injury walking. NeuroRehabilitation.

[81] Arazpour M, Chitsazan A, Hutchins SW, Ghomshe FT, Mousavi ME, Takamjani EE, Aminian G, Rahgozar M, Bani MA (2012). Design and simulation of a new powered gait orthosis for paraplegic patients. Prosthet Orthot Int.

[82] Del-Ama AJ, Gil-Agudo Á, Pons JL, Moreno JC (2013). Hybrid FES-robot cooperative control of ambulatory gait rehabilitation exoskeleton. J NeuroEng Rehabil.

[83] Lerner ZF, Damiano DL, Park H-S, Gravunder AJ, Bulea TC (2017). A robotic exoskeleton for treatment of crouch gait in children with cerebral palsy: design and initial application. IEEE Trans Neural Syst Rehabil Eng.

[84] Bishop L, Stein J, Wong CK (2012). Robot-aided gait training in an individual with chronic spinal cord injury. J Neurol Phys Ther.

[85] Arazpour M, Chitsazan A, Bani MA, Rouhi G, Ghomshe FT, Hutchins SW (2013). The effect of a knee ankle foot orthosis incorporating an active knee mechanism on gait of a person with poliomyelitis. Prosthet Orthot Int.

[86] Arazpour M, Ahmadi Bani M, Samadian M, Mousavi ME, Hutchins SW, Bahramizadeh M, Curran S, Mardani MA (2015). The physiological cost index of walking with a powered knee-ankle-foot orthosis in subjects with poliomyelitis: a pilot study. Prosthet Orthot Int.

[87] Kawasaki S, Ohata K, Tsuboyama T, Sawada Y, Higashi Y. Development of new rehabilitation robot device that can be attached to the conventional knee-ankle-foot-orthosis for controlling the knee in individuals after stroke. In: 2017 International conference on rehabilitation robotics (ICORR); IEEE. 2017. pp. 304–307. 10.1109/ICORR.2017.8009264. 10.1109/ICORR.2017.8009264 28813836

[88] ASIA Store: American Spinal Injury Association - ASIA. Standards for Neurological Classification of SCI Worksheet; 2016. https://asia-spinalinjury.org/wp-content/uploads/2016/02/International_Stds_Diagram_Worksheet.pdf. Accessed 9 Mar 2020.

[89] Platz T, Gillner A, Borgwaldt N, Kroll S, Roschka S (2016). Device-training for individuals with thoracic and lumbar spinal cord injury using a powered exoskeleton for technically assisted mobility: achievements and user satisfaction. BioMed Res Int.

[90] Watanabe H, Goto R, Tanaka N, Matsumura A, Yanagi H (2017). Effects of gait training using the Hybrid Assistive Limb® in recovery-phase stroke patients: a 2-month follow-up, randomized, controlled study. NeuroRehabilitation.

[91] Bach Baunsgaard C, Vig Nissen U, Katrin Brust A, Frotzler A, Ribeill C, Kalke Y-B, León N, Gómez B, Samuelsson K, Antepohl W, Holmström U, Marklund N, Glott T, Opheim A, Benito J, Murillo N, Nachtegaal J, Faber W, Biering-Sørensen F (2017). Gait training after spinal cord injury: safety, feasibility and gait function following 8 weeks of training with the exoskeletons from Ekso Bionics. Spinal Cord.

[92] Baunsgaard C, Nissen U, Brust A, Frotzler A, Ribeill C, Kalke Y, León N, Gómez B, Samuelsson K, Antepohl W, Holmström U, Marklund N, Glott T, Opheim A, Penalva J, Murillo N, Nachtegaal J, Faber W, Biering-Sørensen F (2018). Exoskeleton gait training after spinal cord injury: an exploratory study on secondary health conditions. J Rehabil Med.

[93] Contreras-Vidal JL, Bortole M, Zhu F, Nathan K, Venkatakrishnan A, Francisco GE, Soto R, Pons JL (2018). Neural decoding of robot-assisted gait during rehabilitation after stroke. Am J Phys Med Rehabil.

[94] Nolan KJ, Karunakaran KK, Ehrenberg N, Kesten AG. Robotic Exoskeleton Gait Training for Inpatient Rehabilitation in a Young Adult with Traumatic Brain Injury. In: 2018 40th annual international conference of the IEEE engineering in medicine and biology society (EMBC); IEEE. 2018. pp. 2809–2812. 10.1109/EMBC.2018.8512745. https://ieeexplore.ieee.org/document/8512745/. 10.1109/EMBC.2018.8512745 30440985

[95] Yeung L-F, Ockenfeld C, Pang M-K, Wai H-W, Soo O-Y, Li S-W, Tong K-Y (2018). Randomized controlled trial of robot-assisted gait training with dorsiflexion assistance on chronic stroke patients wearing ankle-foot-orthosis. J NeuroEng Rehabil.

[96] Calabrò RS, Naro A, Russo M, Bramanti P, Carioti L, Balletta T, Buda A, Manuli A, Filoni S, Bramanti A (2018). Shaping neuroplasticity by using powered exoskeletons in patients with stroke: a randomized clinical trial. J NeuroEng Rehabil.

[97] Jayaraman A, O’Brien MK, Madhavan S, Mummidisetty CK, Roth HR, Hohl K, Tapp A, Brennan K, Kocherginsky M, Williams KJ, Takahashi H, Rymer WZ (2019). Stride management assist exoskeleton vs functional gait training in stroke. Neurology.

[98] White HS, Hayes S, White M. The effect of using a powered exoskeleton training programme on joint range of motion on spinal injured individuals: a pilot study. Int J Phys Ther Rehabil. 2015;2015:12. 10.15344/2455-7498/2015/102.

[99] Appelros P, Stegmayr B, Terént A (2009). Women sex differences in stroke epidemiology: a systematic review. Stroke.

[100] Dijsseldonk RB, Rijken H, Nes IJ, Meent H, Keijsers NL (2019). Predictors of exoskeleton motor learning in spinal cord injured patients. Disabil Rehabil.

[101] Lünenburger L, Colombo G, Riener R (2007). Biofeedback for robotic gait rehabilitation. J Neuroeng Rehabil.

[102] Stoller O, Waser M, Stammler L, Schuster C (2012). Evaluation of robot-assisted gait training using integrated biofeedback in neurologic disorders. Gait Posture.

[103] Khoo IH, Marayong P, Krishnan V, Balagtas M, Rojas O, Leyba K (2017). Real-time biofeedback device for gait rehabilitation of post-stroke patients. Biomed Eng Lett.

[104] Tamburella F, Moreno JC, Valenzuela DSH, Pisotta I, Iosa M, Cincotti F, Mattia D, Pons JL, Molinari M (2019). Influences of the biofeedback content on robotic post-stroke gait rehabilitation: electromyographic vs joint torque biofeedback. J Neuroeng Rehabil.

[105] Torricelli D, Pons JL. Eurobench: Preparing robots for the real world. In: International Symposium on Wearable Robotics. Springer; 2018. pp. 375–378. 10.1007/978-3-030-01887-0_72.

[106] Pinto-Fernandez D, Torricelli D, del Carmen Sanchez-Villamanan M, Aller F, Mombaur K, Conti R, Vitiello N, Moreno JC, Pons JL (2020). Performance evaluation of lower limb exoskeletons: a systematic review. IEEE Trans Neural Syst Rehabil Eng.

[107] van Dijsseldonk RB, Rijken H, van Nes IJ, van de Meent H, Keijsers NL (2017). A framework for measuring the progress in exoskeleton skills in people with complete spinal cord injury. Front Neurosci.

[108] Juszczak M, Gallo E, Bushnik T (2018). Examining the effects of a powered exoskeleton on quality of life and secondary impairments in people living with spinal cord injury. Topics Spinal Cord injury Rehabil.

[109] Chang S-H, Afzal T, Berliner J, Francisco GE (2018). Exoskeleton-assisted gait training to improve gait in individuals with spinal cord injury: a pilot randomized study. Pilot Feasibil Studies.

[110] Farris RJ, Quintero HA, Murray SA, Ha KH, Hartigan C, Goldfarb M (2014). A preliminary assessment of legged mobility provided by a lower limb exoskeleton for persons with paraplegia. IEEE Trans Neural Syst Rehabil Eng.

[111] Tsukahara A, Hasegawa Y, Eguchi K, Sankai Y (2015). Restoration of gait for spinal cord injury patients using HAL with intention estimator for preferable swing speed. IEEE Trans Neural Syst Rehabil Eng.

[112] Murray SA, Ha KH, Hartigan C, Goldfarb M. An assistive control approach for a lower-limb exoskeleton to facilitate recovery of walking following stroke. In: IEEE transactions on neural systems and rehabilitation engineering; 2014. 1–1. 10.1109/TNSRE.2014.2346193. 10.1109/TNSRE.2014.2346193 25134084

[113] Li LLL, Ding L, Chen N, Mao Y, Huang D, Li LLL (2015). Improved walking ability with wearable robot-assisted training in patients suffering chronic stroke. BioMed Mater Eng.

[114] Mizukami M, Yoshikawa K, Kawamoto H, Sano A, Koseki K, Asakwa Y, Iwamoto K, Nagata H, Tsurushima H, Nakai K, Marushima A, Sankai Y, Matsumura A (2016). Gait training of subacute stroke patients using a hybrid assistive limb: a pilot study. Disabil Rehabil Assist Technol.

[115] Molteni F, Gasperini G, Gaffuri M, Colombo M, Giovanzana C, Lorenzon C, Farina N, Cannaviello G, Scarano S, Proserpio D, Liberali D, Guanziroli E (2017). Wearable robotic exoskeleton for overground gait training in sub-acute and chronic hemiparetic stroke patients: preliminary results. Eur J Phys Rehabil Med.

[116] Tan CK, Kadone H, Watanabe H, Marushima A, Yamazaki M, Sankai Y, Suzuki K (2018). Lateral symmetry of synergies in lower limb muscles of acute post-stroke patients after robotic intervention. Front Neurosci.

[117] Hassan M, Kadone H, Ueno T, Hada Y, Sankai Y, Suzuki K (2018). Feasibility of synergy-based exoskeleton robot control in hemiplegia. IEEE Trans Neural Syst Rehabil Eng.

[118] Mataki Y, Kamada H, Mutsuzaki H, Shimizu Y, Takeuchi R, Mizukami M, Yoshikawa K, Takahashi K, Matsuda M, Iwasaki N, Kawamoto H, Wadano Y, Sankai Y, Yamazaki M (2018). Use of hybrid assistive limb (HAL®) for a postoperative patient with cerebral palsy: a case report. BMC Res Notes.

[119] Matsuda M, Iwasaki N, Mataki Y, Mutsuzaki H, Yoshikawa K, Takahashi K, Enomoto K, Sano K, Kubota A, Nakayama T, Nakayama J, Ohguro H, Mizukami M, Tomita K (2018). Robot-assisted training using Hybrid Assistive Limb® for cerebral palsy. Brain Dev.

[120] Fisahn C, Aach M, Jansen O, Moisi M, Mayadev A, Pagarigan KT, Dettori JR, Schildhauer TA (2016). The effectiveness and safety of exoskeletons as assistive and rehabilitation devices in the treatment of neurologic gait disorders in patients with spinal cord injury: a systematic review. Global Spine J.

[121] Mehrholz J, Thomas S, Werner C, Kugler J, Pohl M, Elsner B (2017). Electromechanical-assisted training for walking after stroke. Cochrane Database Syst Rev.

[122] Ueba T, Hamada O, Ogata T, Inoue T, Shiota E, Sankai Y (2013). Feasibility and safety of acute phase rehabilitation after stroke using the hybrid assistive limb robot suit. Neurol Medico-chirurgica.

[123] Gagnon DH, Escalona MJ, Vermette M, Carvalho LP, Karelis AD, Duclos C, Aubertin-Leheudre M (2018). Locomotor training using an overground robotic exoskeleton in long-term manual wheelchair users with a chronic spinal cord injury living in the community: Lessons learned from a feasibility study in terms of recruitment, attendance, learnability, performa. J NeuroEng Rehabil.

[124] Spungen AM, Asselin P, Fineberg DB, Kornfeld SD, Harel NY. Exoskeletal-assisted walking for persons with motor-complete paraplegia. NATO Science and Technology Organization; 2013. 15–17 .

[125] Yang A, Asselin P, Knezevic S, Kornfeld S, Spungen A (2015). Assessment of in-hospital walking velocity and level of assistance in a powered exoskeleton in persons with spinal cord injury. Topics Spinal Cord Injury Rehabil.

[126] Benson I, Hart K, Tussler D, van Middendorp JJ (2016). Lower-limb exoskeletons for individuals with chronic spinal cord injury: findings from a feasibility study. Clin Rehabil.

[127] Asselin PK, Avedissian M, Knezevic S, Kornfeld S, Spungen AM (2016). Training persons with spinal cord injury to ambulate using a powered exoskeleton. JoVE.

[128] Shimizu Y, Kadone H, Kubota S, Suzuki K, Abe T, Ueno T, Soma Y, Sankai Y, Hada Y, Yamazaki M (2017). Voluntary ambulation by upper limb-triggered hal® in patients with complete quadri/paraplegia due to chronic spinal cord injury. Front Neurosci.

[129] Contreras-Vidal JL, Grossman RG. Neurorex: A clinical neural interface roadmap for eeg-based brain machine interfaces to a lower body robotic exoskeleton. In: 2013 35th Annual International Conference of the IEEE Engineering in Medicine and Biology Society (EMBC). IEEE; 2013. pp. 1579–1582. 10.1109/EMBC.2013.6609816. 10.1109/EMBC.2013.6609816 PMC3801427 24110003

[130] Benabid AL, Costecalde T, Eliseyev A, Charvet G, Verney A, Karakas S, Foerster M, Lambert A, Morinière B, Abroug N, Schaeffer MC, Moly A, Sauter-Starace F, Ratel D, Moro C, Torres-Martinez N, Langar L, Oddoux M, Polosan M, Pezzani S, Auboiroux V, Aksenova T, Mestais C, Chabardes S (2019). An exoskeleton controlled by an epidural wireless brain-machine interface in a tetraplegic patient: a proof-of-concept demonstration. Lancet Neurol.

[131] Chen B, Ma H, Qin L-Y, Gao F, Chan K-M, Law S-W, Qin L, Liao W-H (2016). Recent developments and challenges of lower extremity exoskeletons. J Orthopaed Transl.

[132] Gorgey AS (2018). Robotic exoskeletons: the current pros and cons. World J Orthoped.

[133] Mekki M, Delgado AD, Fry A, Putrino D, Huang V (2018). Robotic rehabilitation and spinal cord injury: a narrative review. Neurotherapeutics.

[134] Fehlings MG, Tetreault LA, Aarabi B, Anderson P, Arnold PM, Brodke DS, Chiba K, Dettori JR, Furlan JC, Harrop JS (2017). A clinical practice guideline for the management of patients with acute spinal cord injury: recommendations on the type and timing of rehabilitation. Global Spine J.

[135] Sandrow-Feinberg HR, Houlé JD (2015). Exercise after spinal cord injury as an agent for neuroprotection, regeneration and rehabilitation. Brain Res.

[136] Eng JJ, Levins SM, Townson AF, Mah-Jones D, Bremner J, Huston G (2001). Use of prolonged standing for individuals with spinal cord injuries. Phys Ther.

[137] Sale P, Russo EF, Russo M, Masiero S, Piccione F, Calabrò RS, Filoni S (2016). Effects on mobility training and de-adaptations in subjects with Spinal Cord Injury due to a Wearable Robot: a preliminary report. BMC Neurol.

[138] Chisholm AE, Alamro RA, Williams AMM, Lam T (2017). Overground vs. treadmill-based robotic gait training to improve seated balance in people with motor-complete spinal cord injury: a case report. J NeuroEng Rehabil.

[139] Alamro RA, Chisholm AE, Williams AMM, Carpenter MG, Lam T (2018). Overground walking with a robotic exoskeleton elicits trunk muscle activity in people with high-thoracic motor-complete spinal cord injury. J NeuroEng Rehabil.

[140] Goffredo M, Iacovelli C, Russo E, Pournajaf S, Di Blasi C, Galafate D, Pellicciari L, Agosti M, Filoni S, Aprile I, Franceschini M (2019). Stroke gait rehabilitation: a comparison of end-effector, overground exoskeleton, and conventional gait training. Appl Sci.

[141] Brown AR, Martinez M (2019). From cortex to cord: motor circuit plasticity after spinal cord injury. Neural Regener Res.

[142] Curt A, Van Hedel HJA, Klaus D, Dietz V (2008). Recovery from a spinal cord injury: significance of compensation, neural plasticity, and repair. J Neurotrauma.

[143] Miller LE, Zimmermann AK, Herbert WG (2016). Clinical effectiveness and safety of powered exoskeleton-assisted walking in patients with spinal cord injury: systematic review with meta-analysis. Med Dev.

